# Experiences from Two Decades of HTLV-1/2 Testing in a Low-Prevalence Area in Southern Germany, 2004 to 2024

**DOI:** 10.3390/v18080882

**Published:** 2026-08-12

**Authors:** Klaus Korn, Philipp Steininger, Barbara Schmidt, Andrea K. Thoma-Kress, Lennart Forneck, Antje Knöll

**Affiliations:** 1 Harald zur Hausen Institute of Virology, University Hospital Erlangen, Friedrich-Alexander-Universität, Erlangen-Nürnberg, Schlossgarten 4, 91054 Erlangen, Germanyphilipp.steininger@uk-erlangen.de (P.S.); andrea.thoma-kress@uk-erlangen.de (A.K.T.-K.);; 2 Institute of Medical Microbiology and Hygiene, Molecular Microbiology (Virology), University of Regensburg, Franz-Josef-Strauß-Allee 11, 93053 Regensburg, Germany

**Keywords:** human T-cell lymphotropic virus type 1 (HTLV-1), human T-cell lymphotropic virus type 2 (HTLV-2), Germany, diagnostics, antibody detection, proviral load, HTLV-1-associated myelopathy/tropical spastic paraparesis (HAM/TSP), adult T-cell leukemia/lymphoma (ATL)

## Abstract

Human T-cell lymphotropic virus type 1 (HTLV-1) is a neglected pathogen with a heterogeneous global distribution. In low-prevalence areas, diagnostic testing is challenged by limited clinical awareness and the low positive predictive value of screening tests. We retrospectively analyzed 10,891 HTLV-1/2 test results from 7719 patient samples submitted to a specialized laboratory in Germany between 2004 and 2024. Antibody screening had a positive predictive value (PPV) of 31.7% for the Diasorin Murex HTLV I + II assay (39 of 123 reactive samples confirmed) and 34.5% for the Abbott Architect rHTLV-1/2 assay (67 of 194 reactive samples confirmed). Raising the sample/cutoff (s/co) threshold to ≥5 would have increased the PPV to >80%, with only two missed diagnoses per assay. HTLV-1 infection was confirmed in 124 carriers and HTLV-2 infection in three carriers. The vast majority of carriers originated from countries with higher endemicity. A delayed antibody response was observed in HTLV-1 infections via organ transplantation. In four patients with neurological disease, elevated HTLV-1/2-specific antibody indices showed antibody production in cerebrospinal fluid (CSF). HTLV-1 proviral load was measured in 83 carriers, with a median of 20,000 HTLV-1 DNA copies per 10^6^ cells (interquartile range, 4000–119,600). Seventy-four percent (17/23) of high-dose intravenous immunoglobulin (IVIg) preparations contained HTLV-1/2 antibodies, which can lead to false-positive HTLV-1/2 antibody screening results in recipients without underlying infection. Therefore, HTLV-1/2 testing presents two major pitfalls: false-negative results in immunosuppressed patients and false-positive results following IVIg administration.

## 1. Introduction

Human T-cell lymphotropic virus (HTLV) was the first exogenous human retrovirus to be discovered in 1980 [1]. Three additional HTLV types have since been described [2,3,4], but only HTLV type 1 (HTLV-1) and type 2 (HTLV-2) are known to be transmitted from human to human [5]. HTLV-1 and HTLV-2 (HTLV-1/2) primarily infect CD4+ T cells and CD8+ T cells, respectively, and establish chronic infection by integration of proviral DNA in the host DNA (reviewed in [6]). Worldwide an estimated 15 to 20 million individuals are infected with HTLV-1/2 [7,8]. Primary HTLV-1 infection is asymptomatic, but severe disease might develop after many years or decades. HTLV-1-associated myelopathy/tropical spastic paraparesis (HAM/TSP) develops in 0.25 to 3.8% of infected individuals, whereas adult T-cell leukemia/lymphoma (ATL) develops in 1 to 5%. Infectious dermatitis (ID), a severe chronic inflammatory skin disease that predominantly manifests during early childhood, is frequently associated with HAM/TSP [9]. Other inflammatory conditions such as HTLV-1-associated uveitis (HU) have also been described [10,11]. Epidemiological evidence indicates that the adjusted risk of death due to any cause is higher among individuals with HTLV-1 infection than among HTLV-1-negative individuals (reviewed in [12]). In addition, HTLV-1 coinfection has been shown to adversely affect susceptibility, clinical course, and outcomes of a wide range of viral, bacterial and parasitic infections [13]. Infection with HTLV-2 is much less pathogenic and non-oncogenic, although it has rarely been associated with a HAM/TSP-like disease characterized by a lower manifestation rate and a slower progression of symptoms. [14].

HTLV-1 is transmitted via the same routes as human immunodeficiency virus (HIV), namely breastfeeding [15], sexual contact and parenteral exposure (blood transfusions, organ transplants or intravenous drug use) [7]. Unlike HIV, HTLV-1 is transmitted exclusively via infected cells (reviewed in [16]). The worldwide HTLV-1 prevalence has been reported at 0.19%, with a highly heterogeneous geographic distribution characterized by endemic areas in sub-Saharan Africa, South America, the Caribbean, Melanesia, and the Southwestern region of Japan [7], often located adjacent to regions with very low prevalence. The highest prevalence has been reported among the indigenous Australian population [17]. In Europe, HTLV-1 prevalence is much lower ranging from 0 to five cases per 100,000 first-time blood donors. Romania is one European country with a higher prevalence (53 per 100,000 donors) [18,19]. In Germany, there are only few data about the seroprevalence because HTLV-1/2 infection is not a notifiable disease and serostatus is not recorded for blood donors or pregnant women. HTLV-1 infections are extremely rare; the estimated total number of infected individuals was approximately 6000 in 2015 [20]. Consequently, clinicians and laboratory specialists may have limited experience with HTLV-1-associated diseases, leading to challenges in both differential diagnosis and laboratory testing.

In this retrospective study, we analyzed HTLV-1/2 test results collected over a 20-year period in a specialized laboratory located in a low-prevalence region of southern Germany. The objectives were to determine the frequency of HTLV-1/2 infection, evaluate the performance of diagnostic assays, assess the impact of blood product transfusion on HTLV test results, and to describe the distribution of HTLV-1 proviral load in relation to clinical status.

## 2. Materials and Methods

### 2.1. Selection of Samples

The Harald zur Hausen Institute of Virology of the University Hospital Erlangen provides virological diagnostic services for the Nuremberg metropolitan region in southern Germany, which has a population of approximately 3.6 million inhabitants. In addition, the institute served as the German National Reference Center for Retroviruses from 1996 to 2016 and received samples from Germany and other European countries for specialized retrovirus diagnostics.

All samples submitted for HTLV-1/2 testing between 26 October 2004 and 4 April 2024 were included in this study, except for samples submitted for quality control purposes. The study included both cell-free specimens (serum, plasma) for HTLV-1/2 antibody testing and cell-containing specimens (EDTA blood, CSF) for the detection of proviral HTLV-1/2 DNA by polymerase chain reaction (PCR). In many cases, both specimen types were available from the same patient. In addition, we received samples from 23 batches of intravenous immunoglobulins (IVIgs) from various manufacturers.

### 2.2. Detection of Antibodies Against HTLV-1 and HTLV-2

For HTLV-1/2 antibody screening, the enzyme immunoassay Murex HTLV I + II (DaSorin, Saluggia, Italy) and the chemiluminescent immunoassay Architect rHTLV-1/2 (Abbott, Abbott Park, IL, USA) were used to test serum or EDTA plasma samples. Reactive screening results were confirmed using the recomLine HTLV-1 & HTLV-2 IgG Line-immunoassay (Mikrogen, Neuried, Germany), the MPD HTLV BLOT 2.4 (MP Biomedicals, Santa Ana, CA, USA) and the INNO-LIA^®^ HTLV I/II Score Line immunoassay (Fujirebio, Hannover, Germany). Positive immunoblots were used to differentiate between HTLV-1 and HTLV-2 antibodies based on type-specific recombinant antigens.

For patients with simultaneous serum and CSF sampling, the HTLV-1/2-specific antibody index (AI) was calculated by dividing the CSF/serum ratio of HTLV-1/2-IgG concentration by the CSF/serum ratio of total IgG concentration. An AI greater than 1.5 strongly indicates HTLV-1/2-IgG production within the CSF, as the CSF/serum ratio for HTLV-1/2-IgG is physiologically lower than or equal to that for total IgG.

### 2.3. Detection of HTLV-1 and HTLV-2 Proviral DNA

DNA was extracted from cell-containing patient samples with the QIAsymphony^®^ DSP Virus/Pathogen Kit (Qiagen, Hilden, Germany).

A nested PCR was used for the qualitative detection of HTLV-1 and HTLV-2 proviral DNA. In the first PCR, 5 µL DNA was used in a 50 µL reaction volume to amplify a 186 bp PCR product specific for both HTLV-1 and HTLV-2. In the second PCR, 2.5 µL PCR product was used in a 50 µL volume to amplify a 141 bp PCR product with different primer sets specific for either HTLV-1 or HTLV-2 (Table A1). Cycling conditions were 3 min at 95 °C, 90 s at 56 °C, 150 s at 72 °C, 44 cycles of 40 s at 95 °C, 1 min at 56 °C, and 1 min at 72 °C. All samples identified as positive for HTLV-1 or -2 proviral DNA by agarose gel electrophoresis were confirmed by Sanger sequencing of the PCR product (Eurofins Genomics, Cologne, Germany) or by real-time PCR for HTLV-1 proviral load.

HTLV-1 proviral load was quantified by real-time PCR as previously described [21] using 10 µL DNA in a 50 µL reaction volume prepared from a ready-to-use master mix containing HotStartTaq DNA polymerase (QuantiTect Probe PCR Kit, Qiagen). PCR was performed on an Applied Biosystems 7500 Real-Time PCR System (Thermo Fisher Scientific, Dreieich, Germany) under the following cycling conditions: 15 min at 95 °C, followed by 40 cycles of 30 s at 95 °C, 20 s at 55 °C, and 1 min at 65 °C. A serial dilution of the dual-standard plasmid pHTLV-ALB was used to generate the standard curve, and MT-2 cells served as positive controls [21]. All samples, controls and standards were analyzed in duplicates.

### 2.4. Data Acquisition

The primary database exported from the laboratory information system (LIS) contained 12,267 entries, each representing a single HTLV diagnostic test. After excluding quality control samples and entries marked as “not done”, 10,932 entries remained. Of these, 41 originated from 23 batches of IVIg. These results were analyzed separately, leaving 10,891 entries from 7719 samples for the final analysis. Patient characteristics were obtained from the LIS and the clinical information provided by the submitting clinicians. After accounting for follow-up samples from the same patients, serological results from 5421 patients and PCR results from 1163 patients were included in the analyses.

Positive predictive values (PPV) for the antibody screening assays were calculated by dividing the number of true positive results (confirmed by immunoblot and/or detection of proviral DNA) by the total number of reactive and borderline screening results. Additionally, 95% confidence intervals (CIs) for PPV were estimated using the MedCalc Software Ltd. Diagnostic test, Version 23.6.4 [22]. Due to the small sample size and incomplete clinical data, no formal statistical test was performed to compare the HTLV-1 proviral load between the three clinical subgroups (symptomatic, asymptomatic, unknown).

## 3. Results

### 3.1. Results of HTLV 1/2 Antibody Testing

**Figure 1:**
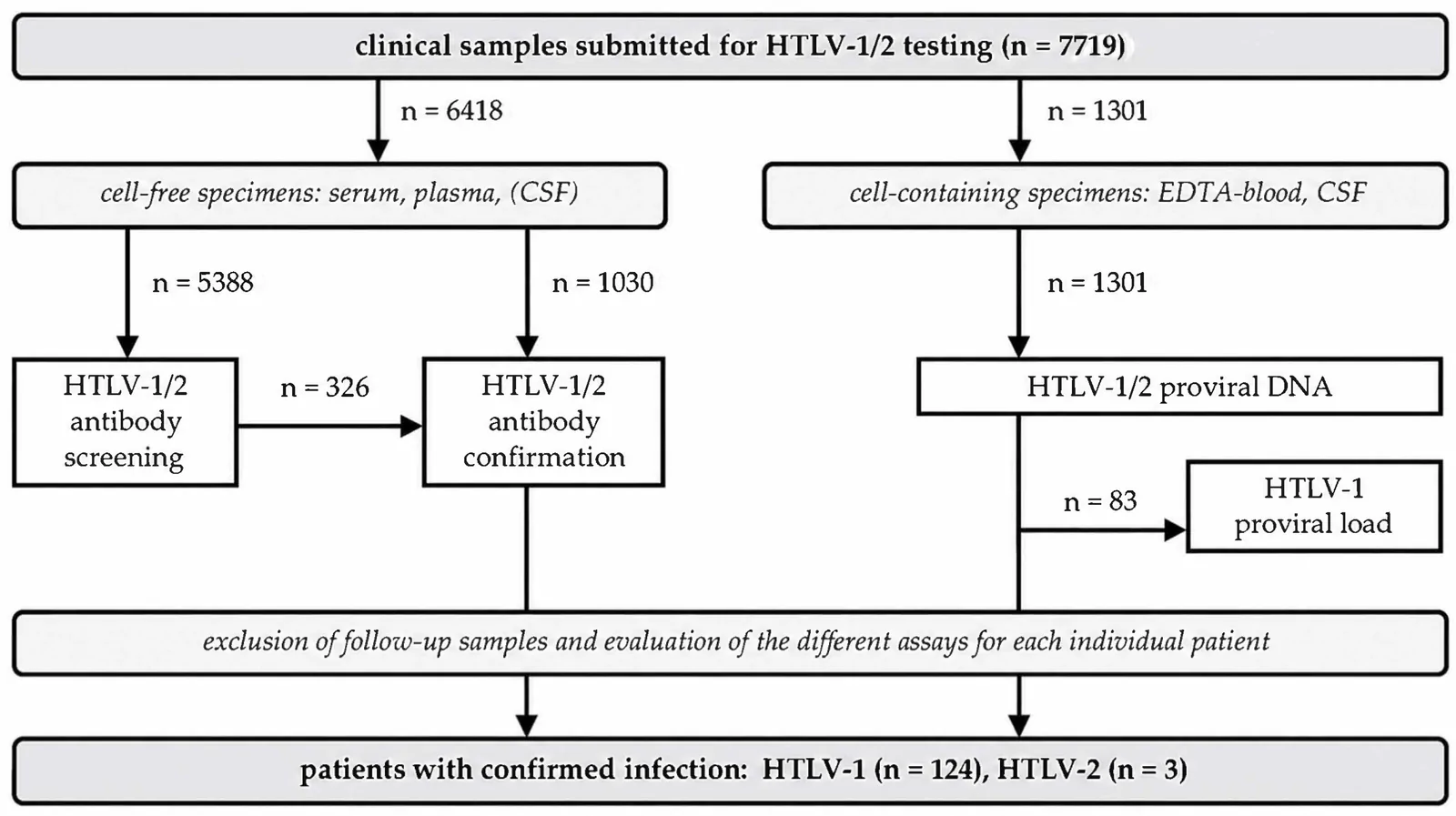
Diagnostic evaluation of 6418 cell-free specimens from 5421 patients and 1301 cell-containing specimens from 1163 patients.

In total, 5388 patient samples were screened for HTLV 1/2 antibodies by the Murex HTLV I + II or the Architect rHTLV-1/2 screening assay (Figure 1; Table 1). Patient samples were serum (n = 4983), EDTA plasma (n = 377) and CSF (n = 28).

Confirmatory immunoblot assays were performed in 1356 samples that either had a reactive or borderline screening test or that were sent directly for antibody confirmation testing. We obtained 1199 negative immunoblot results (88.4%). A total of 66 immunoblots were positive for HTLV-1 (4.8%) and one immunoblot was positive for HTLV-2 (<0.1%). Additionally, 90 immunoblot assays (6.6%) showed indeterminate results: for both HTLV-1 and HTLV-2 (n = 57), for HTLV-1 alone (n = 12) or for HTLV-2 alone (n = 21).

Table 1 shows the results of the screening tests, divided into four levels of reactivity based on the sample cutoff ratio (s/co). Confirmation of the HTLV-positive status was based on positive results in the immunoblot assays in most cases. A few cases with PCR-confirmed HTLV-1 infection and indeterminate or negative immunoblot results were also classified as “confirmed” despite the lack of a positive immunoblot result. Together, the two screening tests identified 106 samples (Murex: 39/2607, Abbott Architect: 67/2931) for which HTLV-1 infection was confirmed during follow-up investigation. To calculate the PPV, samples with borderline screening results were pooled with samples with reactive screening results. This resulted in PPVs of 31.7% (95% CI 27.2 to 36.6%) for the Diasorin Murex HTLV I + II assay (39 confirmed cases among 123 borderline/reactive samples), and 34.5%, (95% CI 30.8% to 38.5%) for the Abbott Architect rHTLV-1/2 assay (67 confirmed cases among 194 borderline/reactive samples).

After accounting for follow-up samples from the same patient, 56 patients had HTLV-1 infection confirmed by immunoblot. Among 90 samples with indeterminate HTLV immunoblots, five samples originated from two patients with PCR-confirmed HTLV-1 infection through organ transplantation and one sample from a patient with PCR-confirmed HTLV-2 infection. In the other 84 indeterminate samples, no evidence of HTLV infection was found.

Antibody testing was performed in 28 CSF samples from 26 patients. Positive results were observed for eight CSF samples. For two of these samples, no corresponding serum sample was available. The remaining six CSF samples, obtained from four patients with confirmed HTLV-1 infection and HAM/TSP, had corresponding serum samples, and total IgG concentrations were available for both CSF and serum This allowed determination of the HTLV-1/2-specific antibody index (AI). As shown in Table 2, all six CSF–serum pairs had elevated HTLV-1/2-specific antibody indices (AI > 1.5), indicating HTLV-specific intrathecal antibody production.

### 3.2. HTLV Antibody Testing of IVIg

In total, 23 high-dose IVIg preparations were screened for the presence of HTLV-1/2 antibodies. Five preparations were nonreactive, one had borderline reactivity and 17 (74%) were reactive in the HTLV-1/2 screening assay. Immunoblot was performed in nine of the reactive samples, and a positive result was obtained for one batch obtained in 2009. The other eight batches were negative in the immunoblot assay.

### 3.3. Detection of HTLV-1 and HTLV-2 Proviral DNA by Nested PCR

Qualitative detection of HTLV-1 and HTLV-2 proviral DNA was performed in 1301 samples which contain or may contain infected T lymphocytes. HTLV-1 DNA was present in 128/1095 EDTA blood samples, 9/194 CSF samples, and 3/4 bronchoalveolar lavage samples. Eight samples (three biopsies, two bone marrow samples, two plasma samples and one vesicle fluid sample) tested negative for HTLV-1 and HTLV-2 proviral DNA. HTLV-1 proviral DNA positive samples originated from 108 patients with proven HTLV-1 infection. HTLV-2 proviral DNA was detected in two EDTA blood samples from two patients.

### 3.4. HTLV-1 Proviral Load

HTLV-1 proviral load was measured in EDTA blood samples from 83 patients with confirmed HTLV-1 infection (Table 3). Fourteen patients had HTLV-1-associated neurological disease or leukemia, whereas seven were asymptomatic carriers. For the remaining 62 patients (74.7%), no clinical information regarding the indication for HTLV testing was provided. The median HTLV-1 proviral load was 20,000 copies per 10^6^ cells (IQR, 4000–119,600) for all carriers and 12,250 copies per 10^6^ cells (IQR, 2200–225,000) among the fourteen patients with known HTLV-1-associated disease.

### 3.5. HTLV-2 Infection

HTLV-2 infection was confirmed in three individuals. Case 1 was a 57-year-old man from Vietnam with chronic myeloid leukemia who was referred after a reactive HTLV-1/2 screening. In our laboratory, the HTLV-1/2 antibody screening was reactive (s/co 48), and the immunoblot was indeterminate. HTLV-2 proviral DNA was detected and confirmed infection. Case 2 was a 53-year-old man from the United States, whose EDTA blood sample was submitted for proviral DNA testing. The sample tested positive for HTLV-2 proviral DNA. Additional evidence for infection was provided by a positive HTLV-2 immunoblot result obtained in 2003 (before the study period). To our knowledge, he remained an asymptomatic carrier. Case 3 was referred to our laboratory because of a reactive HTLV-1/2 antibody screening before umbilical cord blood stem cell collection. In this case, both the HTLV-2 immunoblot and the PCR for HTLV-2 proviral DNA were positive. No additional clinical information was available.

## 4. Discussion

Data on HTLV-1/2 seroprevalence in Germany are limited. Previous studies reported very low prevalence estimates: 0.007% among mothers of 58,747 newborns in 2005 [23], no infections among 100,852 blood donors and 63 intravenous drug users in 2001 [24], and 0.02% among 248,000 blood donors in 1996 [25]. In contrast, our retrospective analysis of clinical samples submitted between 2004 and 2024 identified substantially higher proportions of confirmed HTLV infections: 1.5% (39/2607) with the Murex assay and 2.3% (67/2931) with the Abbott Architect assay. This reflects the highly selected nature of the study population, as samples were predominantly submitted because of suspected HTLV infection or reactive/equivocal screening results obtained elsewhere. Information on ethnicity, country of origin, and clinical presentation was incomplete for many patients and could not be retrieved retrospectively. Nevertheless, based on patients’ names and the origin of samples, including numerous submissions from Romania, we assume that most of the 124 HTLV-1 and three HTLV-2 infections were acquired outside Germany. This observation is consistent with previous reports showing that HTLV infections diagnosed in Germany predominantly occurred in individuals originating from endemic regions [24].

The positive predictive value (PPV) of the screening assays was 31.7% for the Murex assay (39 confirmed among 123 reactive samples) and 34.5% for the Abbott assay (67 confirmed among 194 reactive samples). Restricting positive results to a s/co ≥ 5 would increase the PPV to more than 80% for both assays while causing minimal reduction in sensitivity. Only two HTLV-1 infections would have been missed by each screening assay. None of these samples showed unequivocal HTLV-1 positivity by immunoblot and diagnosis was confirmed by detection of HTLV-1 proviral DNA. Similar conclusions were reported by Luo et al. [26], who found that raising the Murex cutoff to an s/co ≥ 7.0 reduced false-positive results by 95%.

Overall, immunoblot and PCR performed well as confirmatory tests. However, several samples from three patients who acquired HTLV-1 through organ transplantation yielded indeterminate or negative immunoblot results despite PCR-confirmed infection. In addition, one patient with HTLV-2 infection had an indeterminate immunoblot result. Among 62 HTLV-infected patients for whom both immunoblot and PCR results were available, only these four patients had negative or indeterminate immunoblot findings. Conversely, one patient had a positive immunoblot result for HTLV-1 without detectable proviral DNA. Therefore, PCR for proviral DNA proved to be more reliable, particularly in immunosuppressed patients, and the combination of both methods was necessary to detect all cases.

All three recipients who acquired HTLV-1 infection through organ transplantation developed HTLV-1-associated disease: two developed cutaneous T-cell lymphoma within two and three years after transplantation, respectively [27], and one developed HTLV-1-associated myelopathy four years after transplantation [28]. In all three patients, HTLV-1 proviral DNA was consistently detectable after transplantation, whereas seroconversion was delayed by up to two years in screening assays and by up to six years in confirmatory immunoblots. One patient even showed seroreversion (loss of anti-HTLV-1 antibodies) six years after transplantation despite a persistently high proviral load. These observations demonstrate that antibody-based assays may yield false-negative results in patients receiving immunosuppressive therapy and emphasize the importance of PCR testing in this setting.

Conversely, false-positive antibody test results can be caused by the passive transfer of HTLV antibodies via blood products. High-dose IVIg is frequently administered to patients with neurological disorders or suspected autoimmune diseases and can interfere with antibody assays. In an Australian study from a low-prevalence population, nearly 90% of false-positive HTLV screening results were caused by prior IVIg administration [26]. Information on IVIg treatment was unavailable for most reactive samples in our study, but several observations support this mechanism. In 2009, three stem cell recipients showed apparent seroconversion in screening assays, yet immunoblot results were inconclusive and proviral DNA was negative. All three patients had previously received IVIg from the same batch, for which no residual material was available. Analysis of 23 different IVIg batches from several manufacturers demonstrated reactive screening results in 17 (74%). Nine of these batches were also tested by immunoblot, and one yielded a strong HTLV-1 reactivity. In the Murex HTLV screening assay, this batch remained reactive after a predilution of 1:100 (s/co 1.71). Such findings indicate that IVIg may contain sufficient antibody concentrations to generate reactive screening tests and even positive or indeterminate immunoblot results.

Assessment of intrathecal antibody production is crucial in patients with neurological manifestations suggestive of HAM/TSP [29,30]. All four patients with clinically diagnosed HAM/TSP for whom CSF and serum were available showed an elevated specific HTLV-1/2 antibody index (AI). The degree of intrathecal antibody synthesis varied considerably, with AIs ranging from 2.4 to nearly 40.

HTLV-1 proviral load reflects the proportion of infected host cells and has been associated with disease risk. The median HTLV-1 proviral load among 83 infected individuals was 20,000 DNA copies per 10^6^ cells (IQR, 4000–119,600). For most patients, no clinical information was available, as the samples were sent by large laboratories and, according to German data protection regulations, no additional data could be collected retrospectively. As a result, our cohort size precluded a robust statistical analysis of the prognostic value of the HTLV-1 proviral load. Although there is evidence that a high HTLV-1 proviral load is associated with the development of both inflammatory and malignant HTLV-1-associated disease (reviewed in [31]), our study was unable to assess this correlation.

This retrospective study has several limitations. (i) The study examined a selectively biased population that was predominantly referred due to suspected infection, preventing any conclusions regarding overall HTLV-1/2 seroprevalence. (ii) For a large proportion of virus carriers, no clinical and demographic data could be collected; thus, in 75% of cases with proviral load, the clinical context was missing. (iii) Due to the small size of the subgroups (e.g., HTLV-2 carriers, n = 3), statistical tests could not be applied meaningfully. (iv) For the same reason, the proviral load comparisons presented in Table 3 are strictly descriptive and lack formal statistical testing.

## 5. Conclusions

HTLV-1/2 infections are rare in Germany, and most patients originate from areas with higher prevalence. Clinicians must nevertheless consider HTLV-1 infection in neurological and oncological disease, especially in patients from endemic regions. Laboratory diagnosis requires a thorough evaluation of reactive antibody screening results and consideration of potential confounding factors, such as immunosuppression or IVIg administration, which can lead to false-negative or false-positive test results, respectively. Final proof of infection is the detection of integrated proviral DNA in infected lymphocytes.

## Figures and Tables

**Table 1:** HTLV 1/2 antibody screening, all samples combined.

	Murex HTLV I + II			Architect rHTLV—1/2		
	s/co	n (%)	Confirmed Reactivity ^1^ n (%)	s/co	n (%)	Confirmed Reactivity ^1^ n (%)
**nonreactive -**	<0.9	2484 (95.3)	n.d. ^2^	<0.9	2737 (93.4)	n.d. ^2^
**borderline +/-**	0.9–1.1	27 (1.0)	0	0.9–1.1	18 (0.6)	0
**reactive +**	1.11–5.0	53 (2.1)	2/53 (3.8)	1.11–5.0	97 (3.3)	2/97 (2.1)
**reactive ++**	>5.0	43 (1.6)	37/43 (86.0)	>5.0	79 (2.7)	65/79 (82.3)

s/co, sample cutoff ratio; n.d., not done. ^1^ confirmation of specific reactivity by positive immunoblot assay and/or proof of HTLV-1 infection by detection of HTLV-1 proviral DNA in cell-containing samples. ^2^ one nonreactive sample originated from a kidney transplant recipient with detectable HTLV-1 proviral DNA.

**Table 2:** HTLV-1/2 antibodies and HTLV-1 proviral load in CSF from patients with HAM/TSP.

Case	Sex	Age at Testing	CSF/Serum Quotient (×10^−3^)		HTLV-1/2-Specific Antibody Index (AI)	HTLV-1 Proviral Load (Copies/10^6^ Cells)	
			HTLV- IgG	Total IgG		CSF	EDTA Blood
1	f	64	6.0	2.4	2.5	200,000	20,000
2	f	46	11.8	5.0	2.4	230,000	3200
		48	9.6	2.7	3.6	370,000	6300
3	m	54	143.0	3.6	39.7	positive *	n.d.
		57	199.0	5.2	38.3	32,000	50,000
4	m	53	52.6	5.6	9.4	30,000	n.d.

n.d. not done; * not quantified, only qualitative PCR performed.

**Table 3:** HTLV-1 proviral load * in EDTA blood from 83 HTLV-1 carriers.

	HTLV-1-Associated Symptoms Present	Asymptomatic Carriers	Without Clinical Information	All Combined
n (male/female/unknown)	14 (7/7/0)	7 (1/4/2)	62 (24/38/0)	83
Age (years) median (IQR)	47 (35–60)	29 (20–50)	43 (35–57)	45 (34–57)
HTLV-1 proviral load * median (IQR)	12,250 (2200–225,000)	18,000 (2300–100,00)	27,000 (6125–68,250)	20,000 (4000–119,600)

* HTLV-1 DNA copies/10^6^ cells.

## Data Availability

The data presented in this analysis are available on request from the corresponding author.

## Abbreviations

The following abbreviations are used in this manuscript:

HTLV-1	human T-cell lymphotropic virus type 1
HTLV-2	human T-cell lymphotropic virus type 2
HAM/TSP	HTLV-1-associated myelopathy/tropical spastic paraparesis
ATL	adult T-cell leukemia/lymphoma
CSF	cerebrospinal fluid
s/co	sample cutoff ratio
IVIg	intravenous immunoglobulin
IQR	interquartile range
PPV	positive predictive value

## Appendix A

**Table A1:** Primers and probes for the qualitative PCR detection of HTLV-1/2 DNA and for the quantitative PCR detection of HTLV-1 proviral DNA.

	Sequence (5′-3′)	Use	Source
HTLV-1/2-out-s *primer*	CCMTCAAYCCMACAGCTCAGG	HTLV-1/2 *PCR (first round)*	BS
HTLV-1/2-out-as *primer*	GTGGTGRAKYTGCCATCGGGTTTT		
HTLV-1-in-s *primer*	GGACTTGTAGAACGCTCTAATGGCATTC	HTLV-1 *PCR (second round)*	BS
HTLV-1-in-as *primer*	GTGGCAGTTGGTTAACACATTCAGGT		
HTLV-2-in-s *primer*	GCCTGGTCGAGAGAACCAATGGTG	HTLV-2 *PCR (second round)*	BS
HTLV-2-in-as *primer*	TACCACTGGGGTTCATGACATTTAGC		
SK-110 *primer*	CCCTACAATCCAACCAGCTCAG	HTLV-1 *real-time PCR*	[21]
SK-111 *primer*	GTGGTGAGCTGCCATCGGGTTTT		
HTLV-1-pol *probe*	*FAM*-CTTTACTGACAAACCCGACCTACCYATGGA-*TAMRA*		
Alb-s *primer*	GCTGTCATCTCTTGTGGGCTGT	human albumin gene *real-time PCR*	[21]
Alb-as *primer*	AAACTCATGGGAGCTGCTGGTT		
AlbHTLVFAM *probe*	*FAM*-CCTGTCATGCCCACACAAATCTCTCC-*TAMRA*		
